# Trends in Menarcheal Age between 1955 and 2009 in the Netherlands

**DOI:** 10.1371/journal.pone.0060056

**Published:** 2013-04-08

**Authors:** Henk Talma, Yvonne Schönbeck, Paula van Dommelen, Boudewijn Bakker, Stef van Buuren, Remy A. HiraSing

**Affiliations:** 1 VU University Medical Centre, Department of Public and Occupational Health, EMGO+ -Institute for Health and Care Research, Amsterdam, the Netherlands; 2 Netherlands Organization for Applied Scientific Research (TNO), Department of Child Health, Leiden, the Netherlands; 3 Netherlands Organization for Applied Scientific Research (TNO), Department of Statistics, Leiden, the Netherlands; 4 Leiden University Medical Centre, Department of Pediatrics, Leiden, the Netherlands; 5 University of Utrecht, Department of Methodology and Statistics, Utrecht, the Netherlands; CUNY, United States of America

## Abstract

**Aim:**

To assess and compare the secular trend in age at menarche in Dutch girls (1955–2009) and girls from Turkish and Moroccan descent living in the Netherlands (1997–2009).

**Methods:**

Data on growth and maturation were collected in 20,867 children of Dutch, Turkish and Moroccan descent in 2009 by trained health care professionals. Girls, 9 years and older, of Dutch (n = 2138), Turkish (n = 282), and Moroccan (n = 295) descent were asked whether they had experienced their first period. We compared median menarcheal age in 2009 with data from the previous Dutch Nationwide Growth Studies in 1955, 1965, 1980 and 1997. Age specific body mass index (BMI) z-scores were calculated to assess differences in BMI between pre- and postmenarcheal girls in different age groups.

**Results:**

Median age at menarche in Dutch girls, decreased significantly from 13.66 years in 1955 to 13.15 years in 1997 and 13.05 years in 2009. Compared to Dutch girls there is a larger decrease in median age of menarche in girls of Turkish and Moroccan descent between 1997 and 2009. In Turkish girls age at menarche decreased from 12.80 to 12.50 years and in Moroccan girls from 12.90 to 12.60 years. Thirty-three percent of Turkish girls younger than 12 years start menstruating in primary school. BMI-SDS is significantly higher in postmenarcheal girls than in premenarcheal girls irrespective of age.

**Conclusion:**

There is a continuing secular trend in earlier age at menarche in Dutch girls. An even faster decrease in age at menarche is observed in girls of Turkish and Moroccan descent in the Netherlands.

## Introduction

Menarcheal age is the most widely used indicator of sexual maturation and can be used as an indicator of female health, growth and development, and the capacity to reproduce [1]–[5]. Onset of maturation and age at menarche, are influenced by several factors, e.g. genetics, ethnicity, height, weight, body mass index (BMI), and socioeconomic circumstances. [3]; [4]; [6]–[8] Several studies from various developed countries worldwide have shown a systematic decrease in median age at menarche in the past 160 years [1]; [7]; [9]–[14]. In Europe the median age at menarche decreased with 2 to 3 months per decade from 16.5 years in 1840 to about 13.0 years in the 1960s, with 0.5 years variation between countries. [3]; [10]; [11]; [15] In Scandinavian countries the secular trend in age at menarche and stature has stopped, and possibly even reversed in the past three decades [2]; [16]; [17]. Based on three previous Dutch Growth Studies [7]; [18]–[20], Mul et al. concluded that between 1980 and 1997 the secular change towards earlier puberty and age at menarche had stabilized in the Netherlands as well [8]. Several studies suggest a relationship between increasing obesity and decline of median age at menarche [21]; [22]. Others suggest that menarche is accompanied or quickly followed by a rapid increase in body weight [23].

In the Fourth Dutch Growth Study (1997) a correlation between BMI and menarche was only present in normal weight or underweight girls. Beyond a BMI of +1 Standard Deviation Score (SDS) there was no decrease in age at menarche with increasing BMI [8].

The aim of this study is: 1) to assess median age at menarche in the Netherlands, in three ethnic groups (i.e. girls of Dutch, Moroccan and Turkish descent); 2) to assess whether there is a secular trend by comparing data on age at menarche from previous Dutch Growth Studies; and 3) to investigate the differences in BMI-SDS in premenarcheal and postmenarcheal Dutch girls in the 1980, 1997 and 2009 studies.

## Materials and Methods

This study is part of the Fifth Dutch Growth Study, a cross-sectional study performed in 2008/9 in the Netherlands. The study protocol was approved by the Medical Ethical Review Board of Leiden University Medical Centre. Written informed consent was not needed. Measurement of growth in children is part of routine Youth Health Care in the Netherlands. [24]; [25] Data on growth and development were collected in children 0–21 years of age in the Netherlands by trained youth health care professionals. The study design is identical to the Fourth Dutch Growth Study [7], which in turn was based on the Dutch Growth Studies in 1955, 1965, and 1980 [18]–[20]. The design and methods of ‘the Fifth Dutch Growth Study’ have been described elsewhere. [24] Pubertal staging was not registered in the Fifth Dutch Growth Study, as there were no significant differences in onset or pace of secondary sexual characteristics between 1980 and 1997. All Dutch Growth Studies consist of a representative sample of children of Dutch origin. Because of differences in growth between Dutch children and children of Turkish and Moroccan descent, the two largest minority groups in the Netherlands, cohorts were included in the 1997 and 2009 study. [26]; [27] Turkish and Moroccan children were predominantly from the four largest cities in the Netherlands. If the girls, both (biological) parents, or the four (biological) grandparents were born either in Turkey or Morocco, they were defined as first, second or third generation, respectively [28]. To determine age at menarche, each girl aged 9 years and older was asked if she had had her first menstruation (status quo method). BMI was defined as weight in kilograms divided by the square of height in meters, and classified as normal weight, overweight or obesity according to the criteria of the International Obesity Task Force (IOTF). [29] The educational level of the parents was defined as the educational level of the highest educated parent and categorized into low, middle and high level [30].

### Statistical Analyses

Reference curves for age at menarche were obtained from the model with the best fit based on the analysis of variance for each ethnicity and year of study separately. We tested generalized linear and additive logistic models [31] with a binomial family and probit and logit link functions and a smoothing spline fit s() on age with equivalent degrees of freedom ranging between 2 and 4 (default). These models describe the probability of having menarche or not, as a function of age. Differences in these probabilities between year of study and ethnicities were tested with analysis of variance with a model with and a model without an additional independent variable year of study or ethnicity. Previous models were also tested adjusted for BMI-SDS to find out if differences can be explained by differences in BMI-SDS between year of study or ethnic groups. BMI-SDS was calculated using the previously published BMI-for-age references [24]. To test differences in mean BMI-SDS between postmenarcheal and premenarcheal girls within age and year of study, linear regression analyses were performed with BMI-SDS as dependent variable and menarche (two categories), age (linear), year of study (three categories) and the interactions BMI-SDS with age and year of study as independent variables. P-values smaller than 0.05 (two-sided) were considered statistically significant. The statistical analyses were performed in R Version 2.12.0 (The R Foundation for Statistical Computing).

## Results

12,005 children of Dutch origin (6,270 female), 2,582 of Turkish origin (1,267 female), 2,616 of Moroccan origin (1,328 female) were included in the umbrella study (Table 1). Age at menarche in girls 9 years and older was obtained from 2138 girls of Dutch, 282 girls of Turkish and 295 girls of Moroccan origin (Table 2).

**Table 1:** The five Dutch Growth Studies 1955–2009 and their number of participants.

year of execution	year of publication	range of age	number of participants
1955	1960	1–25	16910
1965	1968	0–24	54776
1980	1985	0–21	41870
1997	2000	0–21	20572
2009	2011	0–21	20867

**Table 2:** Trends in distribution of age at menarche for the three ethnicities in the Netherlands.

	n	P10	P50	P90
1955 Du			13.66	
1965 Du	1882	11.80 (11.53–11.93)	13.40 (13.22–13.45)	14.90 (14.61–15.01)
1980 Du	3029	11.69 (11.56–11.85)	13.28 (13.18–13.38)	14.87 (14.73–15.03)
1997 Du	3028	11.77 (11.52–11.82)	13.15 (13.06–13.29)	14.88 (14.69–15.08)
2009 Du	2138	11.47 (11.28–11.67)	13.05 (12.90–13.18)	14.51 (14.34–14.68)
1997 Tu	411	11.3 (10.9–11.6)	12.8 (12.6–13.1)	14.4 (13.9–14.9)
2009 Tu	282	11.2 (10.9–11.6)	12.5 (12.1–12.8)	13.6 (13.2–14.0)
1997 Mo	397	11.5 (11.2–12.0)	12.9 (12.7–13.2)	14.4 (13.9–14.8)
2009 Mo	295	11.2 (10.7–11.7)	12.6 (12.3–13.0)	14.0 (13.6–14.3)

We generated 95% confidence intervals for the P10, P50 and P90 in the final models using the bootstrap percentile method based on 1000 replications. The functions boot() and boot.ci() in R were used.

Du = Dutch girls; Tu = Turkish girls; Mo = Moroccan girls.

**Figure 1:**
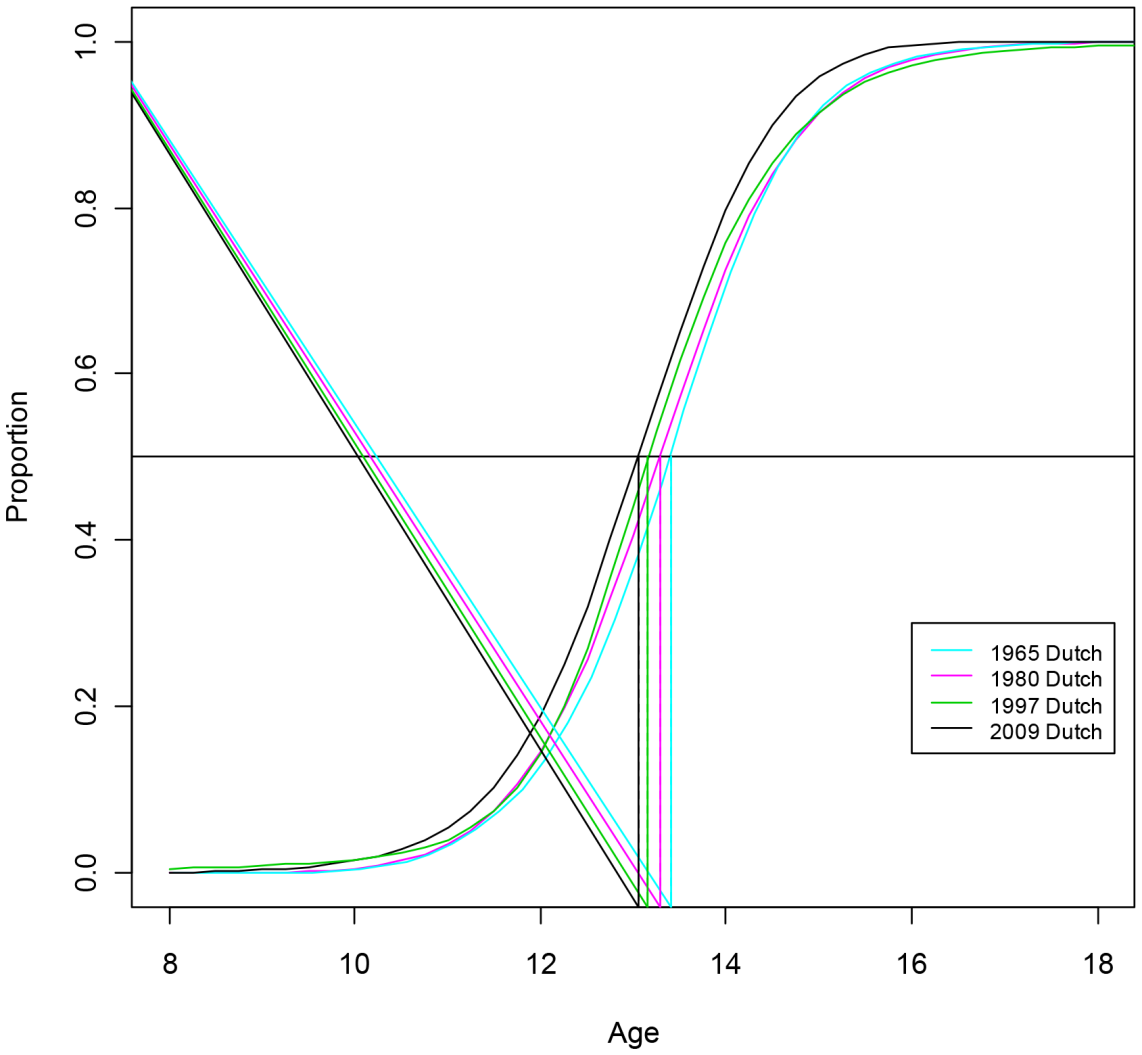
Trends in proportion of menarche in Dutch girls between 1965 and 2009.

**Figure 2:**
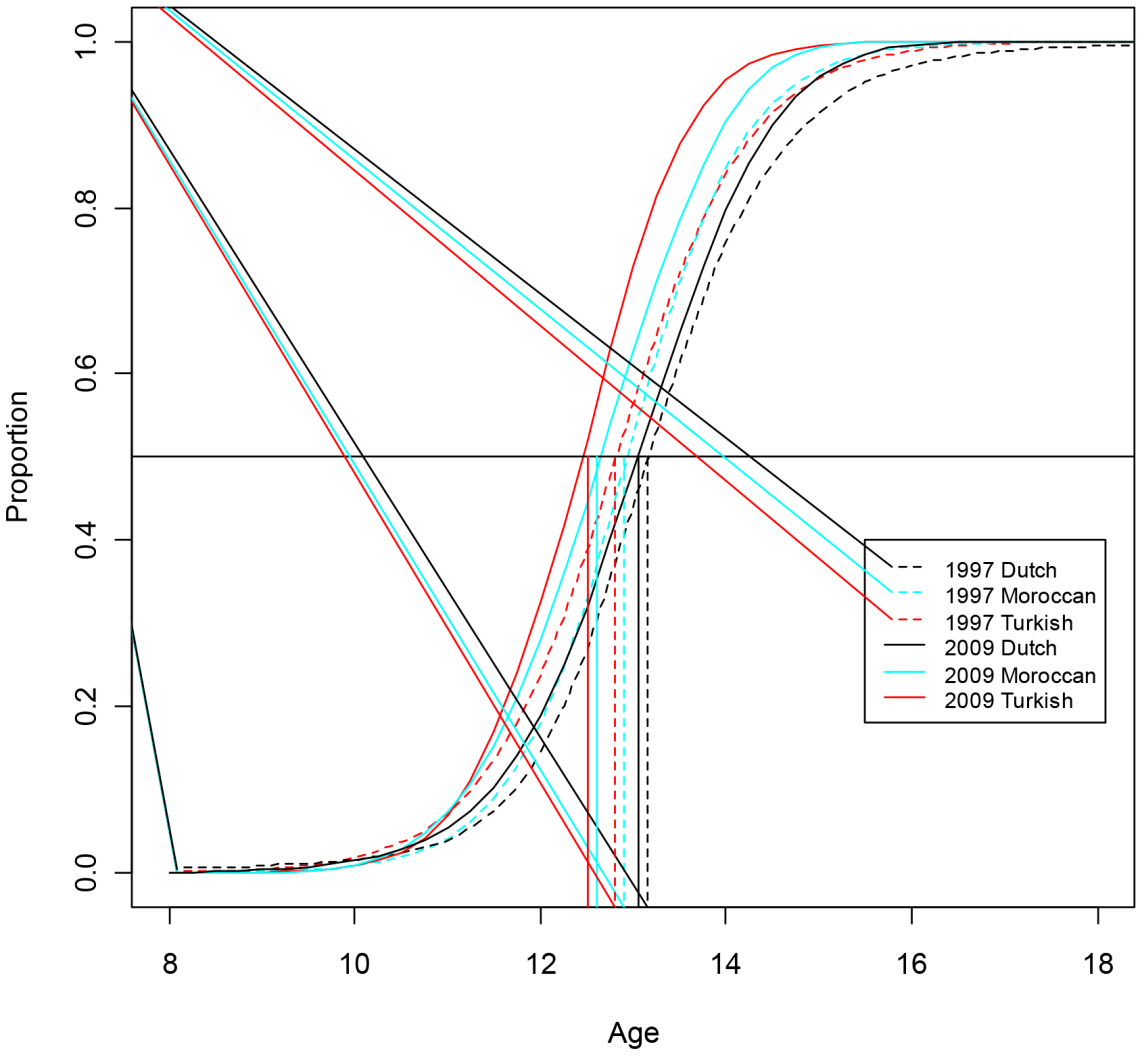
Trends in proportion of menarche in girls from Dutch, Turkish and Moroccan origin between 1997 and 2009.

Table 2 depicts the 10^th^, 50^th^ and 90^th^ centiles and the 95% confidence intervals for age at menarche, specified by ethnicity and data from the previous Dutch Growth Studies (1955, 1965, 1980 and 1997) are added for comparison. The proportion of Dutch girls that reached menarche at different ages is shown in figure 1 for Dutch girls and in figure 2 for Turkish and Moroccan girls. In 2009, median age at menarche in Dutch, Turkish and Moroccan girls was 13.05, 12.50 and 12.60 years, respectively. Median age at menarche occurred at a significantly earlier age than in 1997 for all three ethnicities. In Dutch girls menarche occurred 1.2 months (0.1 years) earlier (p = 0.002; ANOVA); this also holds when adjusted for differences in BMI-SDS (p = 0.004; ANOVA) and in both Turkish and Moroccan girls 3.6 months (0.3 years) earlier (p = 0.02 and p = 0.04, respectively; ANOVA). These differences remain significant after adjustment for BMI-SDS (both p = 0.01; ANOVA). There were significant differences in median age at menarche between Dutch girls and girls of Turkish (p<0.001) and Moroccan (p = 0.007) descent. Similar results were found when adjusted for BMI-SDS (p<0.001 and p = 0.009 respectively; ANOVA). In 1997, only the difference in median age at menarche between Dutch (13.15 years) and Turkish girls (12.80 years) was significant (p = 0.009; ANOVA). However, after adjustment for BMI-SDS, this difference was not significant any more (p = 0.85; ANOVA).

**Figure 3:**
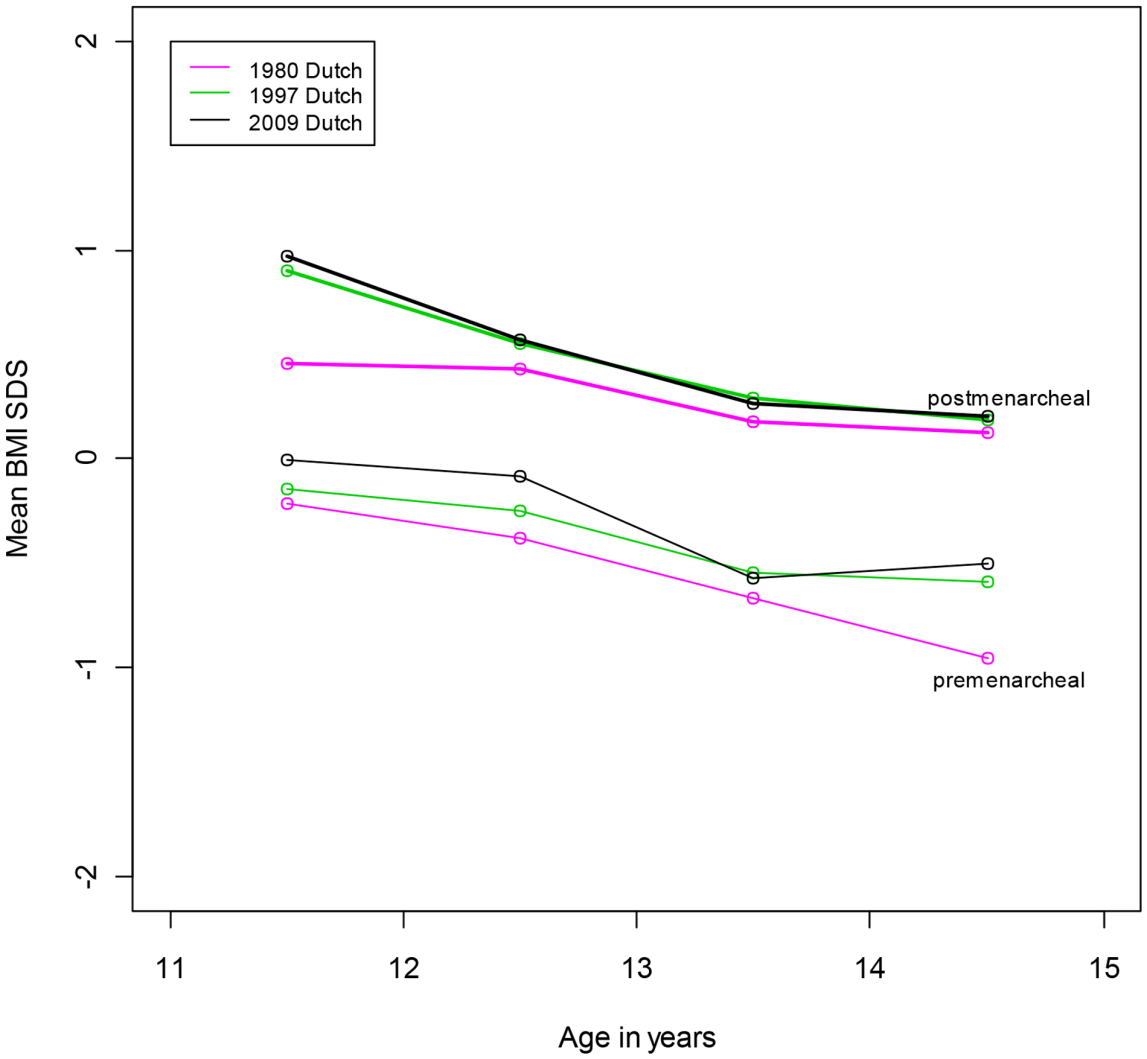
Mean BMI SDS for Dutch premenarcheal and postmenarcheal girls by age in the growth studies in 1980, 1997 and 2009.

There was no significant difference in median age at menarche between girls with high or low educated parents (mothers: 12.9 vs 13.0 years, and fathers 13.2 vs 13.1 years; p = 0.4 and p = 0.7, respectively). Over 90% of parents of the Turkish and Moroccan girls have a low educational level. Figure 3 shows that the mean BMI-SDS in premenarcheal Dutch girls is lower than in postmenarcheal Dutch girls irrespective of age category (data from the Third, Fourth and Fifth Dutch Growth Study combined). There is a significant difference (Δ) of 0.58 in mean BMI-SDS between postmenarcheal and premenarcheal girls, independent of age and year of study (p<0.001). In table 3 is shown that across different ages and studies the differences are similar (Δ 0.66–1.08). The differences (Δ) in the 1980, 1997, and 2009 studies for the age category 13.0–13.9 years are identical (0.84). The proportion of girls of Dutch, Turkish and Moroccan origin that have reached menarche is given by age in table 4. Eight percent of the girls of Dutch, 11% of the girls of Moroccan, and 33% of the girls of Turkish descent have reached menarche before the end of primary school (12 years of age).

**Table 3:** Mean (SD) BMI SDS (according to 1997 references) [49] for premenarcheal (pre-) and postmenarcheal (post-) Dutch girls by age in the 1980, 1997 and 2009 Growth Studies.

Age	1980			1997			2009		
	pre-	post-	Δ	pre-	post-	Δ	pre-	post-	Δ
11.0–11.9	−0.21 (1.01)	0.46 (0.82)	0.67	−0.15 (1.08)	0.90 (0.81)	1.05	0.00 (1.10)	0.97 (0.69)	0.97
12.0–12.9	−0.38 (0.97)	0.43 (0.94)	0.81	−0.25 (1.11)	0.55 (0.79)	0.8	−0.09 (1.14)	0.57 (1.03)	0.66
13.0–13.9	−0.66 (0.92)	0.18 (0.89)	0.84	−0.55 (1.01)	0.29 (0.88)	0.84	−0.57 (1.10)	0.27 (0.88)	0.84
14.0–14.9	−0.95 (1.17)	0.13 (0.89)	1.08	−0.59 (0.96)	0.19 (0.83)	0.78	−0.50 (1.09)	0.21 (1.01)	0.71

Δ = delta: is the difference between mean BMI SDS in premenarcheal and postmenarcheal girls for that age group.

**Table 4:** Proportion of Dutch, Turkish and Moroccon girls with menarche by age in 2009.

Age in years	Dutch 2009	Turkish 2009	Moroccan 2009
	% (n)	% (n)	% (n)
9.0–9.9	1.0 (198)	0 (24)	0 (28)
10.0–10.9	2.7 (256)	0 (28)	7.1 (28)
11.0–11.9	8.7 (196)	33.3 (27)	11.1 (36)
12.0–12.9	32.5 (191)	38.7 (31)	48.5 (33)
13.0–13.9	65.2 (224)	88.6 (35)	77.8 (36)
14.0–14.9	90.9 (263)	100 (36)	95.6 (45)
15.0–15.9	96.9 (224)	100 (44)	100 (30)
≥16.0	100 (125)	100 (24)	100 (28)

## Discussion

Between 1955 and 1965 age at menarche in Dutch girls decreased with 2.5 months per decade. Since 1965, median age at menarche still decreased significantly but with a lower pace of approximately about one month per decade to 13.05 years in 2009. Median menarcheal age in girls of Turkish (12.50 years) and Moroccan (12.60 years) descent was 3.6 months earlier in 2009 than in 1997, a decrease of approximately three months in a decade suggesting a secular trend. In the same period the difference in median age at menarche between Dutch girls and girls from Turkish and Moroccan descent increased with 2.4 months to 6.6 months and 5.4 months, respectively.

### Age at Menarche and Secular Trend in Different Countries Around the World

Is the secular trend in earlier menarche proceeding in other countries as well?

In the United States and Denmark onset of sexual maturation in girls decreased the past decades [4]; [6]; [13]; [21]; [32]. This trend is seen in girls of all different ethnicities (African-American/Caribbean, Hispanics, Indo-Pakistani and Caucasian) [6]; [33]. In contrast, the secular trend in age at menarche stabilized in German girls [3]. In Swedish girls it even reversed. However this may be explained by different study designs making comparison of data difficult [17]. Menarcheal age in girls in Turkey varies in different studies and study populations: 12.36 years in 1975, 13.28 years in 1996, 12.2 and 12.41 years in 2008 and 12.74 years in 2011 [34]–[38]. Bundak et al. as well as Atay et al. found no evidence for a secular trend in age at menarche in girls in Turkey in the last three decades [39]–[41]. Age at menarche in Turkish girls living in Germany in 1985 was 12.90 years, this is comparable to median age at menarche (12.80 years) in girls of Turkish descent in our study 12 years later (1997). [42] Interestingly, in girls from Turkish and Moroccan descent living in the Netherlands age at menarche decreased at a higher pace than it did in Dutch girls. A higher BMI and improved socioeconomic circumstances compared to their country of origin may explain this phenomenon [21]; [26]; [27]; [32]; [43]. To the best of our knowledge, there are no recent data available on menarcheal age in girls in Morocco.

How can we explain the ongoing secular decline in age at menarche?

### Ethnicity and Menarche

Hughes et al. stated that genes remain important in determining the pace of maturation and age of menarche as they account for about 75% of the variance in identical twins, mother/daughter pairs, and girls of the same ethnicity [44]. In industrialized countries girls of African descent have the lowest median age at menarche, followed by Hispanic-Latino and Hindo-Pakistani girls, while Caucasian girls have the highest median age at menarche. [5]; [6]; [13]; [21]; [33]; [45]; [46] Compared to Dutch girls we found significantly lower median age at menarche even after adjustment for BMI-SDS in girls from Turkish and Moroccan origin.

### Socioeconomic Circumstances

Improved health, hygiene, nutrition, housing and employment are assumed to be responsible for most if not all the decline in age at menarche [22]. In classical times until the mid 1950s age at menarche in industrialized countries was lower in girls with high socioeconomic status [47]. Since then the situation reversed and age at menarche in Caucasian girls with low socioeconomic status (SES) compared to girls with high SES became lowest [11]. Studies indicate that girls of African descent in industrialized countries and girls in Turkey with high SES (still) have an earlier age at menarche than girls with low SES [33]; [39]; [48]. Deprivation or malnutrition may be (part of) the explanation. In this study we found no significant differences in median age at menarche between girls from parents with low and high education. In contrast to girls in Turkey the mean BMI-SDS of Dutch children whose parents were low educated was higher in 2009 than those of higher educated parents [24]; [39]; [49].

### Body Mass Index, Weight, Height and Menarche

Several studies suggest that obese girls mature earlier. However, this discussion is controversial [50]–[52] The decline in age at menarche is suggested to be related to the obesity epidemic, but obesity can not solely held responsible, because the secular change was also found in normal weight girls [32]. The link between obesity and age at menarche may be due to a mechanism ensuring that pregnancy will not occur unless there are adequate fat stores to sustain both mother and foetus. [22] Although weight is involved in age at menarche we note that after adjustment for BMI-SDS the decline in median age at menarche remained significant. Because of the cross sectional study design we can not differentiate between co-occurrence and causality. Differences in BMI-SDS in premenarcheal and postmenarcheal girls are previously observed by O’Dea et al., Kaplowitz and others [6]; [21]–[23]. Our data of mean BMI-SDS in premenarcheal and postmenarcheal Dutch girls in different age categories in the 1980, 1997 and 2009 studies show a positive shift in BMI during the maturation process. The differences in mean BMI-SDS between pre- and postmenarcheal girls in 1980, before the obesity epidemic in the Netherlands, are almost identical in 1997 and 2009.

Age at menarche is also thought to be dependent on height. [8] The secular trend in stature stopped decades ago in the United States and Scandinavia[3]; [11]; [53], while in the Netherlands it stopped only recently [25]. The assumption was that the secular trend in age at menarche would stop too, but it did not. Thus, increase of height is no longer (part of) the explanation of the decline in age at menarche in 2009.

### Consequences of Early Menarche for Public Health

Early menarche is associated with psychosocial and health problems [54]; [55]. Already one third (33%) of the girls of Turkish descent in primary schools (up to 12 years of age) is menstruating. The continuing younger age at menarche requires changes in sexual education in primary schools, In addition primary schools have to provide facilities for menstruating girls [15].

Furthermore, adult women with early menarche have a higher risk of breast cancer, metabolic syndrome, depression, and cardiovascular disease [45]; [54]; [56]–[61]. This has profound Public Health implications.

### Strengths and Limitations of the Study

A major strength of the present study is the large study population. The Netherlands have an outstanding tradition of nationwide Growth Studies in 0–21 year old children. This series of growth studies is unique in the world because of the identical design and the national representative samples. By repeating the Growth Study every ten to fifteen years secular trends can be made clear.

A limitation of our study is not having the actual menarche dates of the girls. However the status quo method in a large study population like we used in our study is considered to be even more reliable than the recall method for obtaining menarche dates. A further limitation of the study is the cross sectional character prohibiting conclusions of a causal relationship between age at menarche and BMI.

In the future, monitoring secular trends in children of Dutch, Turkish and Moroccan descent in the Netherlands remains important to assess the differences between ethnicities.

Longitudinal studies are necessary to further elucidate the determinants of age at menarche.
